# Exercise-based modulation of the gut-brain axis: a new direction for neurorehabilitation in Alzheimer’s disease

**DOI:** 10.3389/fnagi.2026.1765016

**Published:** 2026-04-15

**Authors:** ZhaoXie Yu, RongQi Yao, YaNan Li, HanZhang Li, Feng Shen, YanChun Wang, Yao Wang

**Affiliations:** 1 College of Acupuncture-Moxibustion and Orthopaedics, Hubei University of Chinese Medicine, Wuhan, China; 2 Hubei Shizhen Laboratory, Wuhan, China; 3 School of Physical Education and Health, Hubei University of Chinese Medicine, Wuhan, China; 4 School of Nursing, Hubei University of Chinese Medicine, Wuhan, China; 5 College of Chinese Medicine, Hubei University of Chinese Medicine, Wuhan, China

**Keywords:** Alzheimer’s disease, exercise, gut microbiota, gut-brain axis, neuroinflammation

## Abstract

Gut microbiota dysbiosis contributes to the development of Alzheimer’s disease (AD) through gut-brain axis mediated processes, including neuroinflammation, β-amyloid (Aβ) accumulation, tau hyperphosphorylation, disruption of the blood-brain barrier, and progressive cognitive impairment. Exercise, as a non-pharmacological intervention, has been shown to counteract these pathological features by enhancing the production of neuroprotective short-chain fatty acids, reducing systemic and central inflammation, strengthening intestinal barrier integrity, and promoting neuroplasticity. This review integrates preclinical and clinical findings to evaluate the therapeutic potential of exercise in improving cognitive function and attenuating AD pathology, and summarizes key biological mechanisms involving microbiota modulation, short-chain fatty acid (SCFA) metabolism, immune regulation, and gut-brain communication. Current challenges include the limited number of human clinical trials, variability in intervention outcomes, individual differences in responsiveness, and dependence on exercise duration and intensity. Advancing this field will require rigorous longitudinal randomized controlled studies, multimodal therapeutic strategies, and personalized exercise protocols informed by baseline microbiota profiles and genetic risk factors. Although exercise is not curative, it represents an essential component of multitarget interventions and may delay AD progression through modulation of the gut-brain axis.

## 1 Introduction

As a common neurodegenerative disorder, Alzheimer’s disease (AD) is pathologically characterized by senile plaques formed by β-amyloid (Aβ) deposition and neurofibrillary tangles composed of hyperphosphorylated tau protein (Congdon et al., 2023). With the rapid acceleration of global population aging, the incidence of AD continues to rise and has become a major public health concern threatening cognitive function and quality of life in older adults (Rojas-Gualdrón et al., 2024). Although significant advances have been made in understanding the pathological mechanisms of AD in recent years, no effective cure is currently available. Therefore, identifying modifiable pathological pathways and developing non-pharmacological preventive and therapeutic strategies hold substantial clinical and scientific importance (McCullough, 2023).

In recent years, exercise has attracted increasing attention as a safe, accessible, and non-invasive intervention with broad systemic benefits, including the regulation of metabolism, immune function, and neural health (Walzik et al., 2024; Pedersen, 2009). Meanwhile, current pharmacological strategies for AD remain limited in efficacy and often fail to fully address the complex, multifactorial pathophysiology of the disease (Huang et al., 2004; Wang et al., 2017). Accumulating evidence indicates that AD patients frequently exhibit gut microbiota dysbiosis, characterized by reduced microbial diversity, depletion of beneficial bacteria, and enrichment of pro-inflammatory taxa. Such dysbiosis disrupts the production of key metabolites, including short-chain fatty acids (SCFAs), compromises intestinal barrier integrity, and exacerbates neuroinflammation (Jiang et al., 2017; Vogt et al., 2017). Moreover, gut microbes can directly influence Aβ deposition, tau pathology, and neuroplasticity through microbial metabolites, immune modulation, and neuroendocrine pathways, thereby participating in the core pathological processes of AD. It is against this backdrop that exercise, as a non-pharmacological intervention, has shown considerable potential in regulating the gut-brain axis and mitigating AD pathology (Noble et al., 2025). Evidence suggests that exercise can deliberately remodel gut microbiota composition, enhance intestinal barrier function, suppress gut-derived pro-inflammatory cytokines, and promote neurotrophic factor expression and neurogenesis, thereby exerting neuroprotective effects at multiple levels (Dalton et al., 2019; Handajani et al., 2023). Furthermore, in the context of the recently proposed “gut-muscle-brain axis,” exercise may provide an even greater opportunity for multisystem synergistic modulation of neurodegeneration, highlighting its potential as a holistic strategy for AD prevention and intervention.

**Figure 1:**
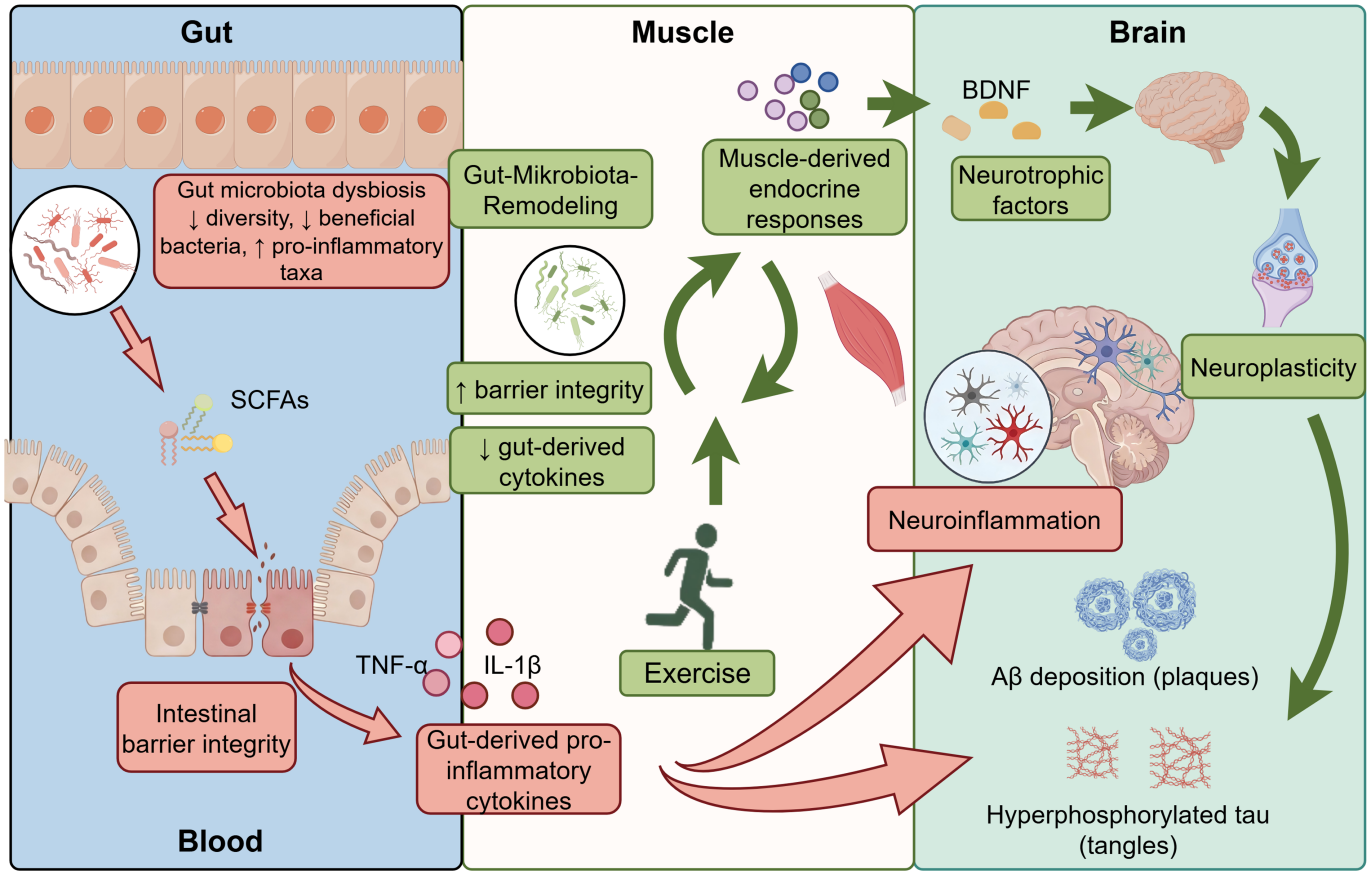
Mechanistic model: exercise-induced microbiota remodeling, barrier reinforcement, and neuroprotection in AD. Graphical figures were created with FigDraw software (https://www.figdraw.com/, accessed on March 19, 2026). Copyright code: USRTIefcce.

Although exercise has emerged as a promising modulator of the microbiota-gut-brain axis, the current evidence base remains uneven. Most mechanistic insights derive from transgenic mouse models, whereas human studies more consistently support benefits for cognition, physical function, and systemic health than for core AD biomarkers. Accordingly, throughout this review we distinguish, where possible, between findings replicated across multiple preclinical studies, preliminary observations derived from single models or single reports, and biologically plausible mechanisms that remain untested in the AD context. We further consider exercise prescription characteristics—including exercise type, training dose, frequency, session duration, and total intervention length—as likely contributors to heterogeneity in microbiota-, inflammation-, and cognition-related outcomes. From this perspective, the present review synthesizes preclinical and clinical evidence on how exercise may influence neuroinflammation, barrier function, microbial metabolites, and AD-related pathology, while also highlighting translational limitations and key directions for future study (Figure 1).

## 2 Search strategy and study selection

This review was conducted as a structured narrative synthesis based on a semi-systematic literature search. PubMed, Web of Science Core Collection, and Embase were searched from database inception to December 2025 using terms related to Alzheimer’s disease, exercise, and the gut microbiota/gut-brain axis, with database-specific adaptations as appropriate. Reference lists of relevant reviews and eligible original studies were also screened manually to identify additional records.

Studies were eligible if they examined exercise interventions in animal or human AD-related settings and reported outcomes related to gut microbiota composition, microbial metabolites, intestinal barrier integrity, inflammatory signaling, cognitive function, or AD-associated pathology. Studies focused exclusively on non-exercise interventions were excluded unless they provided essential mechanistic context. Reviews, editorials, conference abstracts, and studies with insufficient methodological detail were also excluded. After duplicate removal, titles and abstracts were screened, followed by full-text assessment of potentially relevant articles. Screening was performed independently by two authors, and disagreements were resolved through discussion. Given the heterogeneity of study designs, model systems, exercise protocols, and outcome measures, this article should be regarded as a structured narrative review rather than a formal systematic review or meta-analysis.

**Figure 2:**
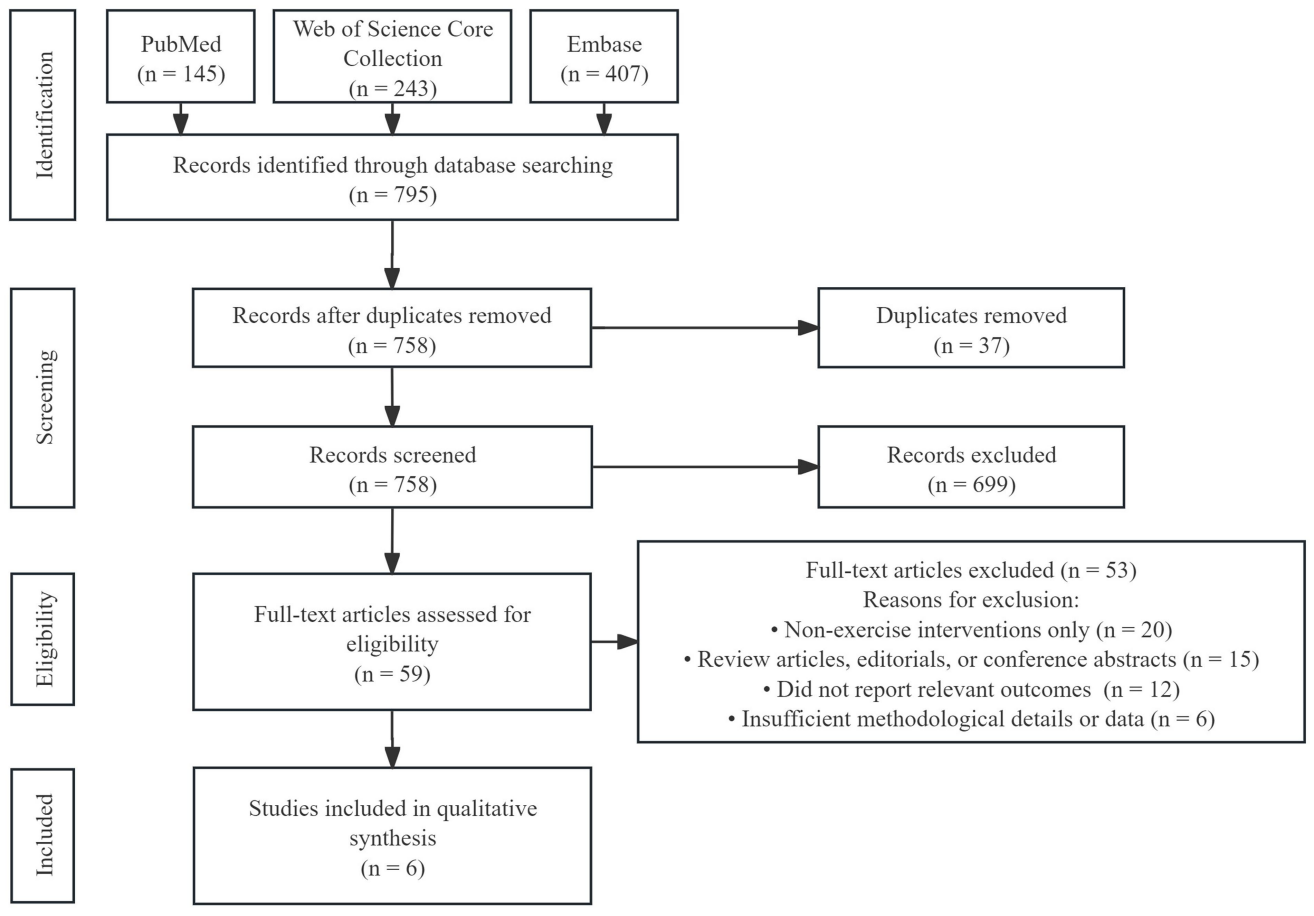
PRISMA flow diagram of literature search and study selection.

A systematic search of PubMed, Web of Science Core Collection, and Embase identified 795 records (145 from PubMed, 243 from Web of Science Core Collection, and 407 from Embase). After 37 duplicates were removed, 758 records underwent title and abstract screening. Subsequently, 59 full-text articles were assessed for eligibility, of which six met the inclusion criteria for qualitative synthesis. The study selection process is shown in the PRISMA flow diagram (Figure 2).

## 3 The role of the gut-brain axis in AD

### 3.1 Gut microbiota dysbiosis in AD

Research evidence indicates that the onset and progression of AD are closely associated with structural and functional disturbances of the gut microbiota. Systematic reviews and meta-analyses have shown that individuals within the AD spectrum generally exhibit reduced gut microbial α-diversity and distinct community clustering, accompanied by characteristic taxonomic imbalances: multiple beneficial SCFA-producing genera (such as Faecalibacterium, Roseburia, and Lachnospira) are diminished, whereas several potentially pro-inflammatory taxa are relatively enriched (Li H. et al., 2024). Mechanistic and translational studies on AD further suggest that the reduction of SCFA-producing bacteria, including those generating butyrate and propionate, in AD and its prodromal stages may weaken intestinal barrier function and immune homeostasis, thereby promoting low-grade inflammation and increasing susceptibility to neuroinflammation (Loh et al., 2024).

Findings from animal research and integrative reviews demonstrate consistent patterns, including reduced abundance of butyrate-producing groups such as Lachnospiraceae and increased load of endotoxin-associated bacteria, a pattern aligned with epithelial barrier impairment, translocation of microbial metabolites, and heightened systemic inflammation (Di Vincenzo et al., 2024; Mishra et al., 2024; Rosendo-Silva et al., 2023). Additional evidence indicates that blood-brain barrier (BBB) integrity can be influenced by the gut-brain axis and gut-derived metabolites. When microbiota dysbiosis coexists with barrier dysfunction, pro-inflammatory mediators and microbe-associated molecular patterns (MAMPs) can enter the circulation and influence the central nervous system microenvironment, thereby exacerbating neuroinflammatory processes (Loh et al., 2024; Fock and Parnova, 2023).

### 3.2 Microbiota-mediated neuroinflammation

Neuroinflammation is a key pathological feature of AD, characterized by sustained activation of microglia and astrocytes, as well as excessive release of pro-inflammatory mediators (Gao et al., 2023). During gut microbiota dysbiosis, intestinal mucosal permeability increases, allowing bacterial products such as lipopolysaccharide (LPS) to enter the circulation and influence the central nervous system through multiple pathways. LPS can activate the Toll-like receptor 4 (TLR4)/nuclear factor kappa-light-chain-enhancer of activated B cells (NF-κB)/NOD-like receptor family pyrin domain-containing 3 (NLRP3) signaling axis in microglia, inducing the expression of pro-inflammatory mediators including interleukin-1 beta (IL-1β) and tumor necrosis factor-alpha (TNF-α), thereby amplifying neuroinflammatory cascades (Skrzypczak-Wiercioch and Sałat, 2022; Li Y. et al., 2024; Li H. et al., 2024).

Conversely, SCFAs such as butyrate, propionate, and acetate exert significant immunoregulatory and anti-inflammatory effects. On one hand, they promote peripheral Treg differentiation and suppress pro-inflammatory pathways via receptors such as free fatty acid receptor 2 (FFAR2) and free fatty acid receptor 3 (FFAR3); on the other hand, SCFAs can cross the BBB through monocarboxylate transporters to directly modulate microglial inflammatory phenotypes and energy metabolism, thereby maintaining central immune homeostasis (Caetano-Silva et al., 2023; Mansuy-Aubert and Ravussin, 2023). In the context of AD-associated gut dysbiosis, the relative abundance of SCFA-producing taxa is decreased, often accompanied by reduced fecal and peripheral SCFA levels. This reduction is thought to weaken the metabolic inhibition of central neuroinflammation (Popescu et al., 2024; Qian et al., 2022).

**Figure 3:**
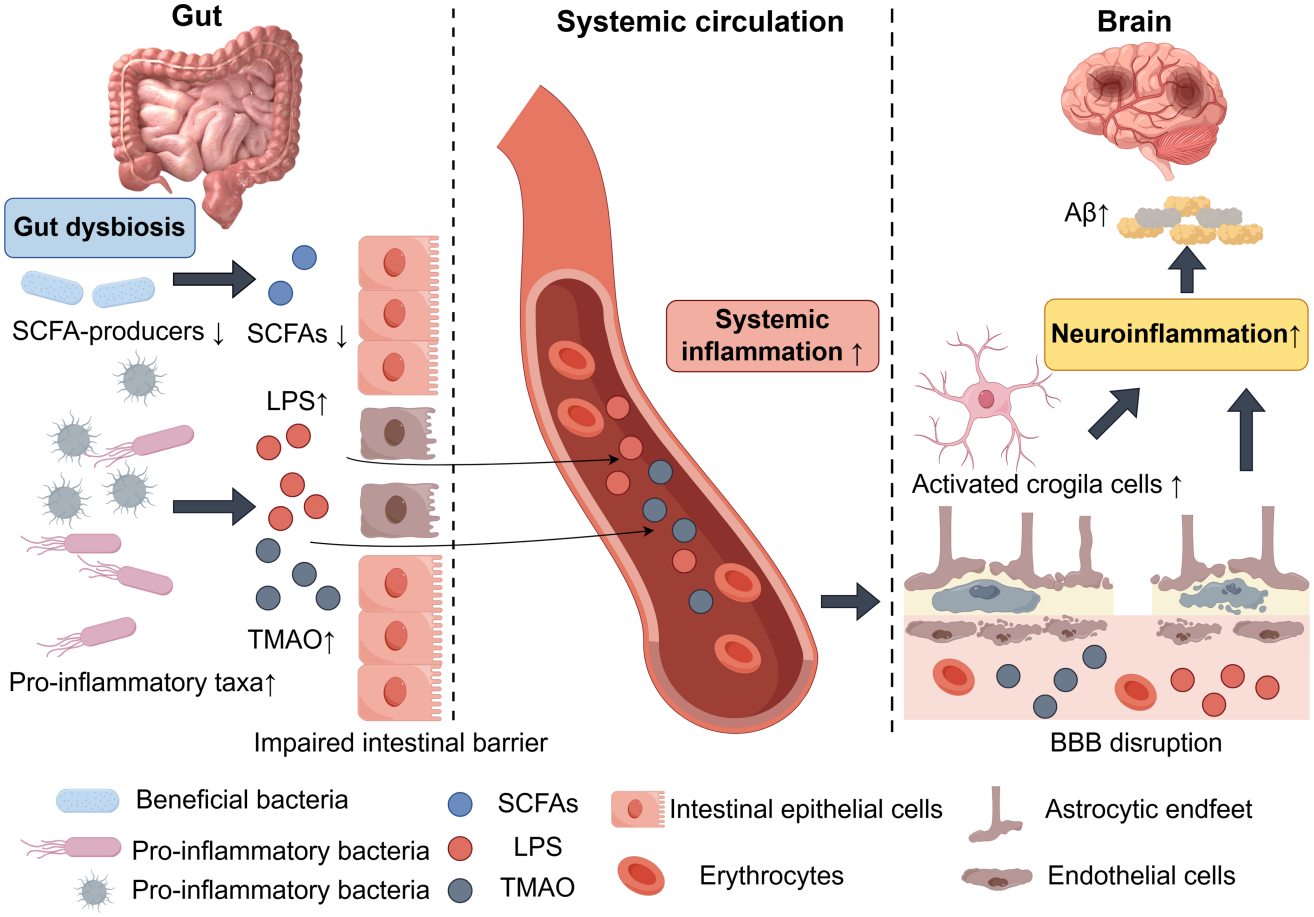
Pathological mechanisms of gut-brain axis dysfunction in AD. Graphical figures were created with FigDraw software (https://www.figdraw.com/, accessed on December 10, 2025). Copyright code: ITUAYb0ccb.

Additionally, gut-derived metabolites such as trimethylamine N-oxide (TMAO) have been associated with cognitive decline and markers of brain structural and small vessel pathology. Recent population and mechanistic studies have highlighted the link between these metabolites, inflammatory responses, and proteinopathies such as Aβ aggregation, suggesting that they may serve as potential risk factors and intervention targets (Figure 3) (Yaqub et al., 2024).

### 3.3 Gut microbiota and AD pathology

While inflammatory signaling provides one plausible route linking the gut to AD, it does not fully explain how peripheral microbial cues align with hallmark proteinopathies. This section therefore summarizes evidence connecting gut-derived factors to Aβ aggregation, tau-related pathways, and broader neuroregulatory changes. Beyond neuroinflammatory pathways, the gut-brain axis participates in core pathological processes of AD through multidimensional mechanisms (Mafe and Büsselberg, 2025). The “pathogen hypothesis” proposes that specific gut microbes can express peptide sequences highly homologous to Aβ. Through molecular mimicry, these peptides elicit adaptive immune responses that cross-react with host proteins, thereby promoting abnormal Aβ aggregation and deposition (Onisiforou et al., 2025). Studies have confirmed that chronic infection or microbial dysbiosis can induce typical AD-like pathological changes in the brain (Kirk et al., 2019; Jin J. et al., 2023; Sait and Day, 2024). Notably, Aβ molecules themselves exhibit broad-spectrum antimicrobial activity, with oligomeric forms effectively disrupting microbial cell membranes, suggesting that Aβ may constitute a critical component of the central nervous system’s innate immune defense (Onisiforou et al., 2025; Kesika et al., 2021). This finding provides a new perspective for understanding Aβ deposition in the AD brain: plaque formation may partially reflect a defensive response of the nervous system to microbial imbalance (Bou Zerdan et al., 2022; Kim et al., 2024).

At the neuroregulatory level, gut microbiota functions as an important metabolic regulatory system capable of biosynthesizing various neuroactive compounds, including neurotransmitters such as serotonin and γ-aminobutyric acid (GABA), as well as small-molecule metabolites derived from tryptophan metabolism (e.g., indole derivatives) with neuromodulatory properties (Kesika et al., 2021). These microbe-produced metabolites may act at the central nervous system through the circulatory system or vagal nerve system, by regulating neuronal excitability, synaptic plasticity and network activity in the brain (Li et al., 2021; Salminen, 2023). Notably, certain microbial metabolites, like indole derivatives, may indirectly influence neuroinflammation and the state of the BBB, by stimulating signaling pathways including the aryl hydrocarbon receptor (AhR) pathway (Salminen, 2023). Though the exact molecular mechanisms between these microbiota-neural pathways in the pathogenesis of AD are yet to be fully clarified, their possibility to have a positive impact on neurotransmitter homeostasis and neural network activity remains a topic of widespread scientific interest and is subject to further examination (Junyi et al., 2025; Yang et al., 2024).

## 4 Effects of exercise on gut microbiota and inflammation

**Figure 4:**
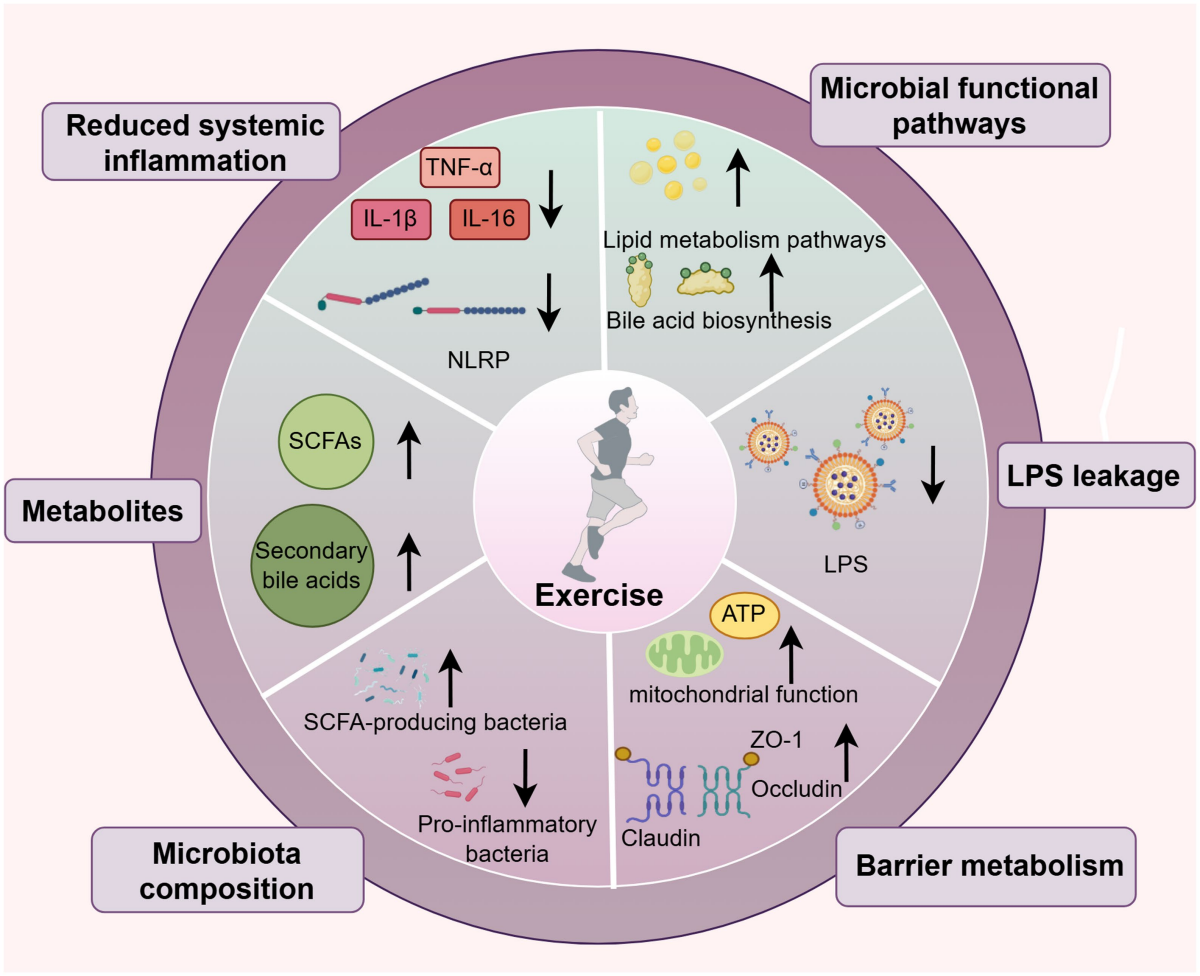
Exercise-induced remodeling of the gut microbiota-systemic inflammation axis in AD. Graphical figures were created with FigDraw software (https://www.figdraw.com/, accessed on December 10, 2025). Copyright code: IITYU3f33d.

At present, the evidence supporting exercise-induced microbiota remodeling in AD is strongest in preclinical models and should not be interpreted as directly equivalent to human therapeutic efficacy. Several findings—including increased microbial diversity, enrichment of selected beneficial taxa, improved barrier-related protein expression, and reductions in LPS-associated inflammatory markers—have been reproduced across animal studies, whereas other observations remain preliminary or model-specific. Human studies provide supportive translational context, but AD-specific clinical microbiome data remain sparse. Against this background, the following sections examine how exercise may modulate gut microbiota composition and systemic inflammation in AD, with particular attention to changes in microbial diversity, enrichment of beneficial taxa and metabolites, reinforcement of intestinal barrier integrity, and suppression of gut-derived inflammatory signaling. These interrelated processes are proposed to attenuate neuroinflammation and contribute to neuroprotection through bidirectional gut-muscle-brain communication (Figure 4). Accordingly, the discussion below distinguishes between comparatively robust preclinical patterns, early translational signals, and mechanisms that remain hypothesis-generating.

### 4.1 Exercise enhances gut microbiota diversity

As a type of systemic intervention, physical exercise has a high regulatory impact on the gut microbial ecosystem (Varghese et al., 2024). Research has demonstrated that in comparison to an inactive lifestyle, routine physical activity is efficient in enhancing gut microbial α-diversity and community organization of the gut niches (Min et al., 2024). This has been found to occur in healthy populations and disease models alike; moderate aerobic activity increases the growth of useful symbiotic bacteria and inhibits colonization by possible pathogenic species (Markus et al., 2025).

As far as AD research is concerned, with treadmill exercise, an animal study showed that 12 weeks of treadmill exercise promoted gut microbial alpha-diversity in an AD model mouse, accompanied by a healthy shift in community composition: relative abundance of opportunistic pathogens declined and neuroprotective taxa became dominant (Yuan et al., 2022; Zhao et al., 2025). This pattern is not limited to APP/PS1 mice. In 3xTg-AD mice, forced treadmill running also induced a symbiotic shift in gut microbial composition, supporting the view that exercise-associated microbiota remodeling can be reproduced across distinct AD models (Jin Y. et al., 2023).

Clinical research supports this tendency. As an example, a randomized-controlled trial that investigated changes in middle-aged overweight or insulin-resistant persons showed that routine aerobic or interval training had a significant impact on the ecology in the gut, including an increased percentage proportion of SCFA-producing Bacteroidetes and a significant decrease in the number of markers associated with plasma endotoxins (Motiani et al., 2020). This provides a theoretical basis for the application of exercise interventions in the prevention and management of AD (Cullen et al., 2023).

### 4.2 Enrichment of beneficial bacteria and metabolites

Existing evidence suggests that exercise interventions may exert relatively consistent effects on certain gut microbial taxa, among which Akkermansia has received particular attention (Aguiar et al., 2024). As a mucosa-associated commensal, Akkermansia is thought to contribute to intestinal barrier maintenance through mucin degradation, and its abundance has been associated with improved barrier integrity and reduced systemic inflammation (Luo et al., 2022). Findings from multiple animal studies, together with limited human evidence, further suggest that regular exercise may increase the relative abundance of Akkermansia (Aguiar et al., 2024; Hintikka et al., 2023).

In AD model mice, treadmill exercise significantly increased intestinal Akkermansia abundance and was accompanied by reductions in serum and brain markers associated with LPS, suggesting that exercise may support gut and blood-brain barrier function while limiting endotoxin translocation (Yuan et al., 2022). A partially convergent finding was reported in 3xTg-AD mice, in which forced treadmill running increased *Akkermansia muciniphila* and reduced Bacteroides species, together with improvements in cognitive performance and AD-like neuropathology (Jin Y. et al., 2023). Taken together, these findings suggest that enrichment of mucosa-associated beneficial taxa may represent a recurring, although not yet universally confirmed, response to aerobic exercise across AD mouse models. However, the AD-specific evidence for Akkermansia remains limited to a small number of preclinical studies, and its translational relevance to human AD should therefore be interpreted cautiously. At present, increased Akkermansia abundance may be regarded as a promising but still preliminary AD-related observation rather than an established clinical biomarker or therapeutic target. More broadly, exercise appears to promote the expansion of beneficial commensals while suppressing potentially unfavorable taxa, thereby shifting the gut microbial ecosystem toward a composition more supportive of host homeostasis (Cullen et al., 2023).

### 4.3 Enhancement of metabolic activity

Aerobic exercise may reshape the metabolic activity of the gut microbiota by promoting SCFA-producing taxa and altering other metabolically relevant pathways (Yu et al., 2019). In AD model mice, functional prediction analysis using the PICRUSt algorithm showed that treadmill exercise enhanced lipid metabolism- and bile acid-related pathways in the cecal microbiota, accompanied by dynamic changes in related metabolites in fecal and tissue samples (Wei et al., 2025). Beyond bile acid metabolism, emerging evidence also implicates trimethylamine N-oxide (TMAO), a gut microbiota-derived metabolite associated with cardiometabolic and neurodegenerative risk. In APP/PS1 mice, exogenous TMAO aggravated cognitive impairment, BBB disruption, gut dysbiosis, and neuroinflammatory responses, whereas voluntary wheel running attenuated these abnormalities and reduced circulating TMAO-related metabolites (Zhang et al., 2023). Consistently, non-targeted HPLC-MS analysis of intestinal contents from APP/PS1 mice showed that long-term aerobic exercise was associated with improved learning ability and broad metabolic reprogramming within the intestinal lumen (Li et al., 2023). Together, these findings suggest that exercise-induced remodeling of gut microbial communities and host–microbe interactions may contribute to metabolic reprogramming in AD-related settings (Yan et al., 2023). Nevertheless, the strength of evidence varies across specific metabolic pathways. Whereas exercise-related shifts in microbial metabolic activity are supported by several preclinical observations, mechanisms involving individual metabolites such as TMAO remain based on limited evidence and should currently be considered preliminary in the AD context.

Notably, many of these microbial metabolites are thought to exert neuroactive and immunoregulatory effects (Yan et al., 2023). For example, certain secondary bile acids may interact with receptors on enterochromaffin cells, potentially modulating vagal signaling and influencing neuroinflammatory states (Hwang and Oh, 2025; Wu et al., 2020), whereas butyrate may promote the release of neurotrophic factors from glial cells and support neuronal survival (Duan et al., 2025). Although the precise signaling pathways remain incompletely understood, current evidence suggests that exercise can beneficially modify gut-derived metabolite profiles, which may in turn contribute to favorable effects on the central nervous system (Wei et al., 2025; Yan et al., 2023).

### 4.4 Suppression of gut-derived inflammation

Another important effect of exercise on the intestinal environment may be the attenuation of gut-derived systemic inflammation (Dmytriv et al., 2024). Sedentary behavior has been associated with chronic low-grade inflammation, partly attributable to increased translocation of bacterial products resulting from impaired intestinal barrier function (Violi et al., 2023). Conversely, regular physical activity has been reported to strengthen the intestinal mucosal barrier and reduce endotoxin translocation into the circulation, thereby potentially lowering systemic inflammatory burden (Motiani et al., 2020).

Preclinical studies further suggest that exercise may enhance intestinal barrier integrity by upregulating key tight junction proteins, including occludin and zonula occludens-1 (ZO-1) (Yuan et al., 2022; Shin et al., 2020). In APP/PS1 mice, Yuan et al. (2022) reported that treadmill training was associated with higher expression of barrier-related proteins in the small intestine and colon, together with improved intestinal permeability relative to sedentary controls. These changes were accompanied by lower serum LPS levels, reduced LPS accumulation in brain tissue, and attenuation of downstream inflammatory signaling (Yuan et al., 2022). Such findings provide a plausible explanation for the reduced microglial activation and lower expression of pro-inflammatory mediators such as IL-1β and TNF-α observed in exercised mice. A partially convergent observation was reported in 3xTg-AD mice, in which forced treadmill running increased BBB-related protein expression alongside favorable microbial shifts, suggesting that exercise-induced gut remodeling may be linked not only to preservation of intestinal barrier function but also to improved neurovascular integrity (Jin Y. et al., 2023).

Exercise may also contribute to a more anti-inflammatory peripheral milieu, as indicated by increased interleukin-10 (IL-10) levels and a higher proportion of regulatory T cells in some experimental settings (Luo et al., 2024). Taken together, current evidence suggests that exercise may mitigate gut-derived inflammatory signaling by strengthening barrier function and reducing endotoxin burden. In this context, the anti-neuroinflammatory effects of exercise in AD may be mediated, at least in part, through reduced production and translocation of peripheral inflammatory stimuli, thereby indirectly limiting central nervous system inflammation and tissue injury (Cutuli et al., 2023). Although these findings form a comparatively coherent preclinical pattern, most evidence for reduced LPS translocation, improved barrier-related protein expression, and downstream anti-inflammatory effects still derives from animal models. Direct confirmation of these mechanisms in human AD remains limited, and caution is therefore warranted when extrapolating these pathways to clinical populations.

### 4.5 “Gut-muscle-brain” axis

The recently proposed concept of the “gut-muscle-brain axis” provides an expanded framework for considering how exercise may modulate the gut-brain axis (Morella et al., 2023). Skeletal muscle, as an important endocrine organ, releases a variety of myokines into the circulation during physical activity, thereby influencing multiple organs and tissues, including the gut and brain (Severinsen and Pedersen, 2020). Research further suggests that skeletal muscle and the gut microbiota communicate bidirectionally through the “gut-muscle axis”: muscle activity may influence the intestinal microenvironment by altering host metabolic and immune status, whereas bioactive metabolites produced by gut microbes, such as SCFAs, may modulate skeletal muscle energy metabolism and functional capacity (Li T. et al., 2024).

Irisin, a representative myokine, has been implicated in hippocampal plasticity and cognitive regulation, and transcriptomic analyses have linked its actions to neurotrophin-related signaling pathways (Islam et al., 2021). Evidence also suggests that irisin may influence gut-brain-axis-related phenotypes by modulating microbial community structure and fecal metabolomic profiles; for example, Fndc5/irisin knockout mice exhibit gut microbial dysbiosis and altered enrichment of microbiota-related metabolic pathways (Liu et al., 2023). Conversely, microbiota-derived metabolites may influence skeletal muscle energetic status and functional reserve (Mishra et al., 2020; Frampton et al., 2020). In C2C12 myotubes, an acetate-propionate-butyrate mixture enhanced insulin-dependent glucose uptake and modulated intracellular glutathione levels, supporting the possibility that SCFAs can directly regulate skeletal muscle cellular metabolism (Otten et al., 2023). Animal studies have also shown that microbiota depletion reduces muscle mass and impairs strength and activity, whereas microbiota reconstitution restores muscle mass and improves strength; supplementation with microbial metabolites such as SCFAs can partially reverse these phenotypes, suggesting a causal contribution of microbiota-derived metabolites to muscle function (Lahiri et al., 2019). Mechanistically, SCFAs may regulate skeletal muscle glucose-lipid metabolism and mitochondrial biogenesis through FFAR2/FFAR3 and energy-sensing pathways such as AMPK-PGC-1α, although tissue specificity, dosage, and exposure duration introduce uncertainty (Frampton et al., 2020).

Clinical studies provide some translational support for gut-muscle interactions, although not yet specifically in AD populations. Older adults with sarcopenia often show alterations in gut microbial structure and function associated with reduced muscle mass and impaired physical performance (Kang et al., 2021). In individuals with metabolic syndrome, fecal microbiota transplantation has been shown to improve host insulin sensitivity, largely through enhanced peripheral glucose disposal, implying that shifts in the gut metabolic ecosystem may contribute to peripheral energy regulation (Vrieze et al., 2012). However, direct effects on muscle mitochondrial function, exercise adaptation, and downstream neurodegenerative outcomes still require confirmation in specifically designed randomized controlled studies.

Taken together, these findings support the biological plausibility of bidirectional signaling between skeletal muscle and the gut microbiota. However, direct evidence linking specific myokines—particularly irisin—to gut microbiota modulation in the context of AD remains limited. At present, the best-supported evidence concerns the independent effects of exercise on myokine signaling and on microbiota-derived metabolites, whereas direct causal links between these systems in AD remain largely inferential. The gut-muscle-brain axis should therefore be viewed as a hypothesis-generating framework supported by convergent biology, rather than a fully validated mechanistic pathway in AD.

### 4.6 Substrate-specific peripheral metabolic pathways across exercise modalities

Distinct exercise modalities may recruit different regulatory routes along the gut-brain axis, leading to modality-specific effects on AD-related pathology. Current evidence suggests that aerobic exercise is frequently associated with broad remodeling of gut microbial community structure and bile acid-related metabolic signatures. In early-stage APP/PS1 mice, sustained treadmill training markedly reshapes the gut microbiota. Integrating fecal metabolomics with functional prediction further indicates perturbations in bile acid-associated pathways, accompanied by improved cognitive performance in behavioral assays (Wei et al., 2025).

Notably, hydrophilic bile acids such as tauroursodeoxycholic acid (TUDCA) can cross the BBB and demonstrate neuroprotective activity across neurological models, including attenuation of endoplasmic reticulum stress and restoration of proteostasis (Khalaf et al., 2022; Song et al., 2024). Taken together, these findings support the hypothesis that aerobic exercise may partially exert central benefits through bile acid-dependent mechanisms, potentially involving TUDCA or other specific bile acid species. In contrast, resistance training may preferentially engage lactate-centered metabolic routes and their downstream microbial transformations. Lactate generated during high-intensity contractions can enter the intestinal lumen and be utilized by specific taxa (e.g., Veillonella), which convert lactate into propionate and other metabolites (Scheiman et al., 2019). Propionate, in turn, acts as an immunometabolic signal that may influence microglial metabolic programming and inflammatory responses under Aβ-related stimulation, thereby modulating microglial reactivity within the AD milieu (Gold et al., 2024; Colombo et al., 2021; O’Riordan et al., 2022). These observations suggest that resistance training could contribute to neuroprotection, at least in part, via metabolically driven immunomodulation.

**Figure 5:**
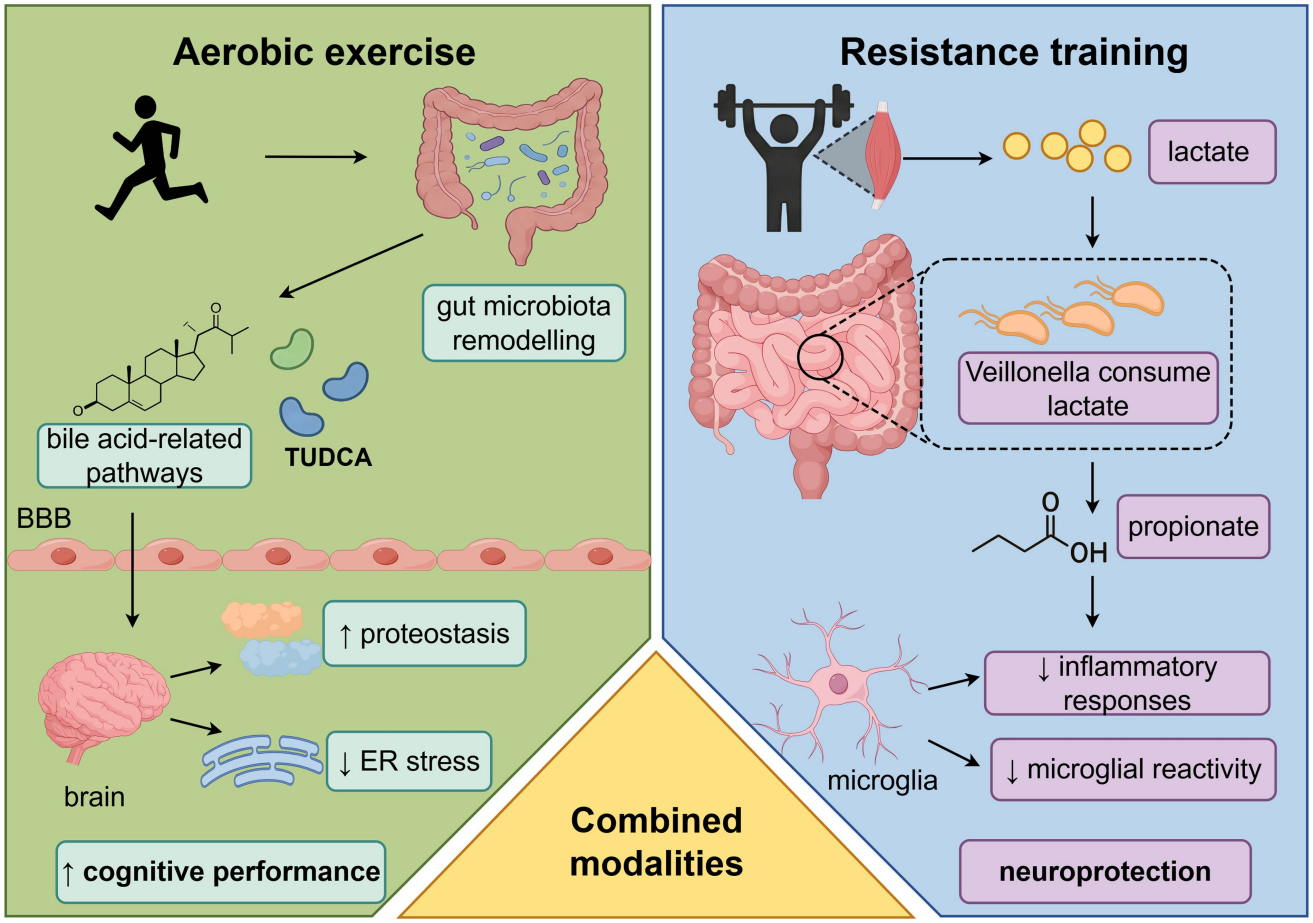
Proposed schematic of aerobic versus resistance training pathways along the gut-brain axis in AD. Graphical figures were created with FigDraw software (https://www.figdraw.com/, accessed on March 21, 2026). Copyright code: IOTYI555f5.

Importantly, aerobic- and resistance-linked pathways are not mutually exclusive. In clinical and rehabilitation contexts, combining modalities according to individual tolerance and therapeutic goals may allow simultaneous targeting of bile acid- and lactate-related peripheral metabolic nodes, thereby enhancing the robustness and durability of gut-brain-axis regulation (Figure 5).

### 4.7 Exercise prescription characteristics across studies

Because exercise responsiveness may depend on protocol characteristics, a more structured comparison of intervention design across studies is warranted. Table 1 summarizes the included AD-related studies according to exercise type and intervention duration, together with their reported gut-related and cognitive or neuropathological outcomes. Overall, the available evidence is dominated by aerobic exercise paradigms, particularly treadmill-based interventions in APP/PS1 and 3xTg-AD mouse models. Across these studies, exercise was most commonly associated with favorable changes in gut microbial composition, metabolite-related pathways, barrier-related markers, and selected cognitive outcomes, although the magnitude and specificity of these effects varied across models and protocols. By contrast, direct comparisons across exercise modalities and doses remain limited, and detailed reporting of training frequency, session duration, and workload progression is not always consistent. As a result, current evidence supports the general importance of exercise prescription characteristics, but does not yet allow robust conclusions regarding the optimal exercise dose or modality for modulating the gut-brain axis in AD.

**Table 1:** Exercise protocols and key gut-related and cognitive outcomes in included AD-related studies.

Subject/Model	Exercise type	Protocol duration	Key gut-related outcomes	Key cognitive/Neuropathological outcomes	References
APP/PS1 transgenic mice	Treadmill aerobic exercise	12 weeks	↑ *Akkermansia*; ↓ *Allobaculum*; improved barrier-related protein expression; ↓ LPS burden	↓ IL-1β and TNF-α; improved spatial learning and memory	Yuan et al. (2022)
APP/PS1 transgenic mice (early-stage AD)	Motorized treadmill aerobic exercise	20 weeks	↑ *Ileibacterium* and *Faecalibaculum*; enriched bile acid- and lipid metabolism-related pathways; altered metabolite profiles	Delayed cognitive decline; improved Morris water maze performance	Wei et al. (2025)
3xTg-AD mice	Forced treadmill aerobic exercise	20 weeks	↑ *Akkermansia muciniphila*; ↓ *Bacteroides*; favorable microbiota remodeling	Improved cognition; reduced AD-like neuropathology	Jin Y. et al. (2023) and Jin J. et al. (2023)
APP/PS1 transgenic mice	Voluntary wheel running	12 weeks, continuous access	↓ TMAO-related metabolites; improved gut dysbiosis/BBB-related changes	Improved cognition; reduced neuroinflammation and AD-related pathology	Zhang et al. (2023)
APP/PS1 transgenic mice	Long-term aerobic exercise	Long-term intervention	Intestinal metabolic reprogramming based on non-targeted HPLC-MS	Improved learning ability	Li et al. (2023)
AD model mice and wild-type mice	Treadmill exercise	12 weeks	Altered gut microbiota composition; model-dependent microbial responses	Supports exercise-related microbiota modulation in AD-related settings	Zhao et al. (2025)

BBB, blood-brain barrier; LPS, lipopolysaccharide; TMAO, trimethylamine N-oxide; HPLC-MS, high-performance liquid chromatography-mass spectrometry; Aβ, amyloid-β. Outcomes are summarized conservatively from the original reports. Protocol duration is presented where clearly available from the source study.

## 5 Mechanisms of exercise on the gut-brain axis in AD

**Figure 6:**
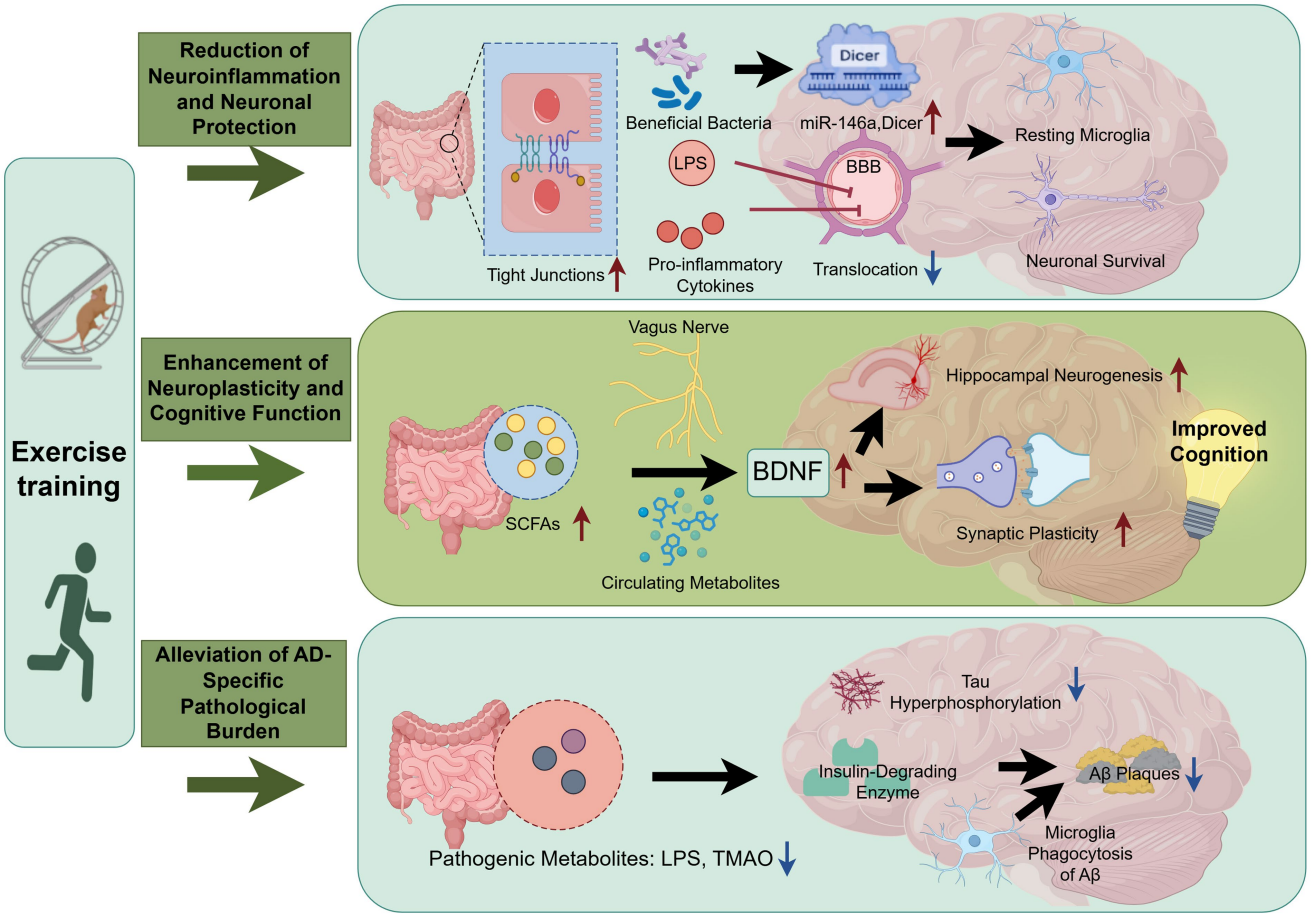
Exercise confers neuroprotective effects in AD through remodeling of the gut-brain axis. Graphical figures were created with FigDraw software (https://www.figdraw.com/, accessed on December 10, 2025). Copyright code: SYUAYcd3aa.

The effects of exercise on AD are neuroprotective, as this impact is instigated by altering the gut-brain axis, which is linked with various physiological processes, and these include immune regulation, metabolic equilibrium, and neuroplasticity (Figure 6).

### 5.1 Reduction of neuroinflammation and neuronal protection

Physical activity intervention can successfully reduce gut-derived neuroinflammatory effects by enhancing intestinal protective capabilities and altering microbiota composition, which is essential in the prevention and treatment of AD and intestinal disorders (Yuan et al., 2022). Chronic neuroinflammation not only directly impedes neuronal functionality but also facilitates abnormal deposition of Aβ and hyperphosphorylation of tau, whereby pathological amplification results in a cascade (Chen and Yu, 2023). Physical training reduces the translocation of inflammatory mediators such as LPS to the central nervous system, effectively interrupting this vicious cycle (Yuan et al., 2022). Experimental studies have shown that voluntary wheel running significantly decreases the number of activated microglia in the hippocampus of APP/PS1 transgenic mice, suppresses neuroinflammatory responses, enhances neuronal survival, and increases synaptic density (Zhang et al., 2022). Further research indicates that exercise upregulates Dicer expression, altering brain microRNA (miRNA) profiles, which may contribute to reduced Aβ accumulation and delayed AD progression (Dungan et al., 2020). Among these, miR-146a, a negative feedback regulator of neuroinflammation, has been independently shown to inhibit excessive inflammatory responses and improve AD-like pathology and cognitive function (Fan et al., 2020; Mai et al., 2019). These findings suggest that exercise may modulate neuroinflammation via epigenetic regulatory pathways (Dungan et al., 2020; Kopczyńska and Kowalczyk, 2024). Notably, changes in miRNA expression may be partially mediated by the gut microbiota. Certain microbial metabolites, such as butyrate, have been shown to influence host miRNA expression, playing a key role in the “microbiota-miRNA-inflammation” regulatory network (Casado-Bedmar and Viennois, 2022; Ney et al., 2023). Wang et al. (2025) systematically proposed the “exercise-microbiota-microRNA” interactive axis, emphasizing that the dynamic balance among these three components jointly determines neuroinflammatory levels. Hence, exercise interventions inhibit neuroinflammatory regulation and prevent neuronal inflammatory damage through various gut microbiota-mediated routes, which is a vital feature through which physical practice postpones AD pathological progression.

### 5.2 Promotion of neuroplasticity and cognitive function

The beneficial effects of exercise on the central nervous system plasticity are well established, encompassing mechanisms such as enhanced synaptic plasticity, facilitation of long-term potentiation (LTP), and induction of hippocampal neurogenesis (Guo et al., 2022). These structural neural changes are closely associated with improvements in cognitive function (Choi et al., 2018). Notably, the gut microbiota plays a critical regulatory role in mediating exercise-induced neurotrophic effects (Delgado-Ocaña and Cuesta, 2024). Brain-derived neurotrophic factor (BDNF), a key mediator of exercise-induced cognitive enhancement, shows markedly increased expression following physical activity (Ben Ezzdine et al., 2025). Gut microbiota integrity is essential for normal BDNF expression: germ-free mice or mice subjected to antibiotic-induced microbiota depletion exhibit significantly reduced baseline hippocampal BDNF levels and attenuated exercise-induced upregulation (Nicolas et al., 2024). Mechanistically, bioactive metabolites derived from gut microbiota, such as SCFAs, can promote BDNF synthesis via vagal nerve signaling and endocrine pathways (Bravo et al., 2011; O’Riordan et al., 2022).

At the neurogenesis level, recent studies have confirmed that exercise can counteract microbiota dysbiosis-induced inhibition of hippocampal neurogenesis. Nicolas et al. (2024) demonstrated that antibiotic-induced dysbiosis significantly impairs the formation of new neurons and associated cognitive functions in the hippocampus of rats, whereas voluntary wheel running effectively reverses this pathological process. This finding underscores the pivotal role of the gut-brain axis in exercise-mediated enhancement of neuroplasticity.

In the context of AD, exercise interventions exhibit dual regulatory benefits: on one hand, they optimize microbiota composition and increase levels of beneficial metabolites such as SCFAs, relieving inhibition of hippocampal neurogenesis (Yuan et al., 2022); on the other hand, they promote the survival and functional integration of new neurons through upregulation of neurotrophic factors and network activity (Choi et al., 2018). Animal experiments confirm that long-term exercise training significantly increases the number and survival of hippocampal newborn neurons in AD model mice and improves cognitive performance. When neurogenesis is inhibited via genetic or pharmacological approaches, the cognitive benefits of exercise are markedly diminished, indicating that stimulation of neurogenesis and synaptic remodeling constitutes a key mechanism by which exercise enhances cognitive function in AD (Choi et al., 2018).

### 5.3 Attenuation of AD-specific pathological burden

In addition to modulating systemic inflammation and neuroplasticity via the gut-brain axis, aerobic exercise may also influence core AD pathology more directly. Evidence is stronger and more consistent for reductions in amyloid-β (Aβ) burden, whereas effects on tau pathology appear comparatively indirect (Xu et al., 2023).

For Aβ pathology, a study in TgCRND8 mice reported that 5 months of voluntary wheel running significantly reduced amyloid plaque load in key regions, including the prefrontal cortex and hippocampus, and decreased soluble Aβ1–40 and Aβ1–42 (Adlard et al., 2005). Mechanistically, exercise can remodel the gut microbiota in AD models (e.g., enriching taxa such as Akkermansia) and improve intestinal barrier integrity (Yuan et al., 2022). It may also increase the abundance of SCFA-producing bacteria; for instance, the butyrate-producing genus Faecalibacterium rises after exercise (Wei et al., 2025). These adaptations may provide dual protection. First, barrier restoration limits translocation of pro-inflammatory endotoxins such as LPS into the circulation, thereby reducing peripheral inflammatory input and creating a more permissive immune environment for Aβ clearance (Harach et al., 2017). Second, elevated beneficial metabolites (e.g., butyrate) may cross the BBB, dampen pro-inflammatory microglial activation, and enhance microglial phagocytic clearance of Aβ (Okumura et al., 2021; Fock and Parnova, 2023; Caetano-Silva et al., 2023).

For tau pathology, prevailing models suggest that exercise acts mainly by attenuating peripheral inflammation and thereby reducing aberrant activation of tau-related kinase pathways. In APP/PS1 mice, treadmill training reshapes the gut microbiota and reduces pathological LPS translocation, which may lessen central inflammatory burden (Yuan et al., 2022). Peripheral LPS can exacerbate tau hyperphosphorylation by activating brain CDK5 and related kinase networks (Kitazawa et al., 2005). Consistent with this mechanism, long-term treadmill training lowers hippocampal phosphorylated tau in 3xTg-AD mice while increasing p-Akt and p-GSK-3β, providing molecular evidence that exercise can mitigate tau phosphorylation burden (Kim et al., 2025).

Exercise may also facilitate Aβ removal by enhancing intracerebral clearance pathways. Human neuroimaging studies indicate that long-term habitual physical activity is associated with improved cerebrospinal fluid-interstitial fluid exchange and increased meningeal lymphatic drainage (Yoo et al., 2025). In parallel, animal studies report that high-intensity interval training (HIIT) restores and preserves the polarized distribution of astrocytic aquaporin-4 (AQP4) in AD-like models, accompanied by reduced cerebral Aβ burden and attenuated tau hyperphosphorylation (Feng et al., 2023). Together, these findings support the notion that, in specific contexts, exercise may enhance Aβ clearance by improving brain fluid dynamics and drainage.

## 6 Clinical studies and neurorehabilitation applications

### 6.1 Neurorehabilitation practice

Exercise interventions have accumulated evidence for application to the whole range of cognitive disorders (Erickson et al., 2022). Epidemiological literature illustrates that there is a persistent correlation between sustaining increased exercise levels during middle and late adulthood and the prevention of cognitive decline (Su et al., 2022). In AD high-risk groups, an inverse relation between long-term, regular aerobic exercise and cognitive trajectories is shown, as well as a correlation between long-term, regular aerobic exercise and a decrease in systemic inflammation and improvement in gut microbiota composition, which may indicate a multi-system protective effect (Dieckelmann et al., 2023; Ramos et al., 2022). Large-scale longitudinal studies, while not demonstrating a significant advantage of aerobic training over active controls, establish the critical value of consistent physical activity in maintaining cognitive stability (Baker et al., 2025). Accordingly, international guidelines now include regular physical activity as a core recommendation for the prevention and management of cognitive decline (Noguchi-Shinohara et al., 2023).

In rehabilitation practice, exercise interventions should extend beyond cognitive maintenance to comprehensively address multi-dimensional outcomes, including cardiopulmonary fitness, muscle strength and balance, mood, sleep, and activities of daily living. Their advantages include a broad safety window and ease of integration with multidisciplinary programs (Chodzko-Zajko et al., 2009). Tailored programs are necessary for patients at different disease stages: for those with mild impairment, moderate-intensity aerobic training combined with resistance exercises and mind–body integration practices, delivered under supervised conditions to enhance adherence, is recommended (Li F. et al., 2024; Yu et al., 2024). For moderate to severe patients, functional maintenance strategies should be applied using low-to moderate-intensity, segmented exercise plans with appropriate safety monitoring systems (Stuckenschneider et al., 2020). Exercise prescriptions should be individualized, specifying training parameters and progression criteria, and integrated into a rehabilitation management system with regular assessments and behavior change strategies to allow continuous adjustment and optimization.

### 6.2 Clinical evidence and biomarker heterogeneity

Clinical evidence from randomized controlled trials and cohort studies indicates that the benefits of exercise are often not temporally aligned with changes in core AD biomarkers. In an early 6-month randomized controlled trial, high-intensity aerobic training improved selected cognitive domains in individuals with mild cognitive impairment, including executive function, and favorably affected indices of glucose metabolism and endocrine function. However, changes in peripheral Aβ markers, such as plasma Aβ42, were modest and did not reach statistical significance (Baker et al., 2010). Likewise, a large intervention study in cognitively unimpaired older adults with established cerebral amyloid deposition found that long-term aerobic exercise did not significantly alter the rate of amyloid accumulation (Vidoni et al., 2021). Collectively, these findings suggest that, in the presence of confirmed pathology, exercise may more readily improve brain function, brain structure, and global health outcomes, whereas slowing or reversing core pathological biomarkers may require longer exposure or more refined participant stratification. Overall, preclinical studies more consistently support the capacity of exercise to mitigate Aβ-related pathology. Clinical studies more robustly demonstrate benefits for cognition and broader brain health outcomes, but effects on core biomarkers such as Aβ vary across populations, intervention characteristics, and assessment windows. Future trials should systematically incorporate multidimensional biomarker panels within the AT(N) framework and include peripheral mechanistic measures, such as intestinal barrier integrity and systemic inflammatory markers. Participants should also be stratified according to key modifiers, including APOE genotype, baseline microbiome profiles, and cardiorespiratory fitness, to improve biological interpretability and reproducibility.

## 7 Discussion

The review critically incorporates emerging evidence on the mechanisms and advancement of exercise regimens in regulating the gut-brain axis in preventing and treating AD. AD is a systemic, multifactorial disorder. The neuroprotective effects of exercise mediated through the gut-brain axis are unlikely to arise from a single pathway. Instead, they likely reflect coordinated changes in the gut microbial ecosystem, microbial metabolites, intestinal barrier integrity, and neuroimmune responses. Available evidence indicates that exercise more consistently improves gut microbiota features and endotoxin burden (Motiani et al., 2020), whereas changes in core pathological biomarkers such as Aβ and tau are often modest or undetectable in clinical studies (Vidoni et al., 2021). Accordingly, exercise is better conceptualized as an intervention that optimizes the peripheral milieu and thereby indirectly buffers central pathological burden, rather than one that directly targets a single pathological endpoint. Consistent with this view, exercise more frequently attenuates Aβ-related pathology or inflammatory phenotypes in preclinical models (Adlard et al., 2005), while clinical studies more reliably demonstrate benefits for cognitive outcomes but show limited or variable effects on Aβ and tau (Baker et al., 2010). Variability across studies may reflect differences in baseline pathological status, intervention dose (duration and intensity), adherence, biomarker measurement methods, and follow-up windows. As summarized in Table 1, this heterogeneity also extends to exercise type, intervention duration, and the level of detail used to report training protocols, which complicates direct comparison across studies. Therefore, integrative analyses linking peripheral alterations, central responses, and functional outcomes may better clarify how exercise confers benefit. Building on the gut-brain axis literature, this review further proposes a gut-muscle-brain axis framework. By integrating evidence for bidirectional interactions between myokines and gut-derived metabolites, this framework positions skeletal muscle not only as an effector of movement but also as a potential regulator of peripheral homeostasis. However, the direct causal role of specific myokines, particularly irisin, in microbiota-mediated modulation of AD remains to be established. Accordingly, the gut-muscle-brain axis should currently be viewed as a biologically plausible and hypothesis-generating framework rather than a fully validated mechanistic pathway in AD.

In terms of intervention parameters, existing evidence suggests that moderate- to high-intensity aerobic exercise may be more effective than low-intensity training in improving inflammatory status and neuroplasticity-related outcomes, although the balance between efficacy, tolerability, and safety requires careful consideration. In addition to this, personal variation is also a serious element that affects the results of the intervention. The microbiome-immune axis may also be shaped by genetic predisposition (e.g., APOE4 genotype), metabolic comorbidities, and other contextual factors that influence responsiveness to exercise interventions. Among these contextual factors, diet is likely to be particularly important.

Exercise effects on the gut-brain axis are unlikely to be independent of diet. Habitual dietary patterns shape microbial ecology, metabolite production, redox balance, and inflammatory tone, all of which may influence responsiveness to exercise. In particular, Mediterranean-style dietary patterns have been linked to gut microbiota modulation and neuroprotective mechanisms relevant to AD, whereas extra virgin olive oil, a key component of the Mediterranean diet, has been associated with antioxidant and anti-inflammatory effects relevant to exercise recovery and systemic resilience (Mafe and Büsselberg, 2025; Perrone and D’Angelo, 2025). These observations suggest that dietary context may modify exercise-induced changes in microbial remodeling, barrier function, and peripheral immunometabolism. However, direct evidence for coordinated exercise-diet-microbiota mechanisms specifically in AD remains limited, and future studies should consider diet not only as background context but also as a measurable modifier and potential co-intervention.

Several additional confounders may modify both gut microbiota composition and responsiveness to exercise in AD. These include age, sex, hormonal status, body mass index, baseline cardiorespiratory fitness, medication exposure—particularly antibiotics and proton pump inhibitors—baseline microbiota composition, and metabolic comorbidities such as obesity or insulin resistance. Sex is particularly relevant as a biological variable because microbiome structure, hormonal milieu, immune signaling, and exercise adaptation all show sex-dependent variation. Moreover, male and female AD models may exhibit differential responses to comparable exercise regimens, suggesting that sex may moderate both microbiota-related and neurological outcomes. Baseline microbiota composition may act as an important moderator of exercise efficacy by influencing whether exercise preferentially enriches SCFA-producing taxa, reduces endotoxin-associated taxa, or produces only minimal detectable change. Future studies should therefore include tighter reporting and, where feasible, stratified analyses based on sex, diet, medication exposure, and baseline microbiota characteristics.

Nevertheless, several limitations should be acknowledged. The same caution applies to the proposed gut-muscle-brain axis, for which direct evidence linking specific myokines to microbiota-mediated AD modulation remains limited. First, there remains a substantial translational gap between animal and human evidence. Many of the mechanistic pathways discussed in this review—including exercise-related increases in Akkermansia, reduced LPS translocation, improved barrier-related protein expression, and modulation of specific gut-derived metabolites—are supported primarily by APP/PS1 or 3xTg-AD models. Although these findings are biologically informative, species differences, model-specific pathology, controlled housing conditions, and relatively standardized exercise exposure limit direct extrapolation to clinical AD. Accordingly, mechanistic findings from animal studies should be interpreted as plausible pathways and testable targets rather than definitive evidence of equivalent effects in patients. Second, high-quality human evidence, particularly randomized controlled trials with AD-specific microbiome endpoints, remains limited. In addition, heterogeneity in microbiome profiling methods, exercise prescriptions, participant characteristics, and outcome assessment likely contributes to variability across studies. Finally, the current literature is weighted heavily toward aerobic exercise, whereas the effects of other modalities, particularly resistance training, on the gut-brain axis in AD remain insufficiently characterized.

Future studies should integrate single-cell sequencing, spatial transcriptomics, and multi-omics analyses to resolve region- and cell type-specific responses to exercise and their epigenetic regulatory networks. Longitudinal cohorts with repeated measures may further define the temporal coupling between microbial metabolomic trajectories and cognitive decline, aiding the identification of intervention windows and biologically informed stratification. Clinically, combining gut metabolomic profiles and endotoxin burden with neuroimaging and fluid biomarkers will be important for narrowing the gap between mechanistic insight and translational relevance.

## 8 Conclusion

The intrinsic relationships between exercise, gut microbiota and brain health are explained systematically in this review, and it is proposed that involving exercise interventions in a multi-faceted approach to AD may result in a multi-target intervention strategy, where both brain nervous system and gut microenvironment are regulated in a synergistic manner. That can be expected as through increasing the understanding of the molecular mechanisms through which exercise acts on the gut-brain axis, as well as through the optimization of exercise prescriptions on an individualized case basis, exercise therapy will most likely assume an even more significant place in AD prevention and treatment. Overall, exercise as a modulator of the microbiota-gut-brain axis is one of the highly promising approaches to AD management. Further studies in this line of research will, in addition, not only broaden our knowledge on the multifaceted pathophysiology of AD, but also potentially offer effective and integrative preventive and therapeutic strategies, which can, in the final stage, enhance clinical outcomes and quality of life of patients.
